# Overview of the Nucleic-Acid Binding Properties of the HIV-1 Nucleocapsid Protein in Its Different Maturation States

**DOI:** 10.3390/v12101109

**Published:** 2020-09-29

**Authors:** Assia Mouhand, Marco Pasi, Marjorie Catala, Loussiné Zargarian, Anissa Belfetmi, Pierre Barraud, Olivier Mauffret, Carine Tisné

**Affiliations:** 1 Expression Génétique Microbienne, UMR 8261, CNRS, Université de Paris, Institut de Biologie Physico-Chimique (IBPC), 75005 Paris, France; assia.mouhand@gmail.com (A.M.); marjorie.catala@ibpc.fr (M.C.); pierre.barraud@cnrs.fr (P.B.); 2 Laboratoire de Biologie et de Pharmacologie Appliquée (LBPA), UMR 8113 CNRS, Institut D’Alembert, École Normale Supérieure Paris-Saclay, Université Paris-Saclay, 4, Avenue des Sciences, 91190 Gif sur Yvette, France; marco.pasi@ens-paris-saclay.fr (M.P.); loussine.zargarian@ens-paris-saclay.fr (L.Z.); anissa.belfetmi@hotmail.fr (A.B.)

**Keywords:** HIV-1, nucleocapsid, nucleic acids, RNA, RNA-binding protein, NMR

## Abstract

HIV-1 Gag polyprotein orchestrates the assembly of viral particles. Its C-terminus consists of the nucleocapsid (NC) domain that interacts with nucleic acids, and p1 and p6, two unstructured regions, p6 containing the motifs to bind ALIX, the cellular ESCRT factor TSG101 and the viral protein Vpr. The processing of Gag by the viral protease subsequently liberates NCp15 (NC-p1-p6), NCp9 (NC-p1) and NCp7, NCp7 displaying the optimal chaperone activity of nucleic acids. This review focuses on the nucleic acid binding properties of the NC domain in the different maturation states during the HIV-1 viral cycle.

## 1. Introduction

**Figure 1:**
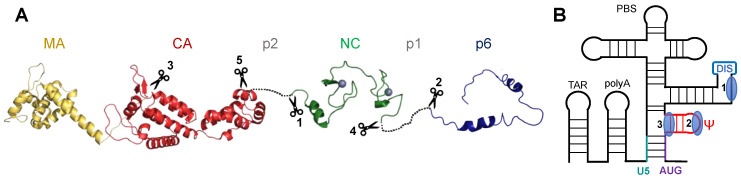
Organization of the Gag protein and of the 5′-leader gRNA: (**A**) Juxtaposition of structures solved for the different domains of Gag (MA: PDB 1UPH, CA: PDB 3GV2, NC: PDB 1A1T, p6: PDB 2C55), the scissors indicating the cleavage sites by the viral protease and the numbers indicating the order of cleavage; (**B**) Secondary structure of the 5′-leader gRNA in the dimer promoting conformation (AUG: *gag* translation start codon, DIS: Dimerization Initiation Site that promotes genome dimerization, PBS: Primer Binding Site for initiation of RT, polyA: polyAdenylation signal, Ψ: packaging signal hairpin, TAR: *trans*-Activator Response region that stimulates transcription). The main binding sites of NCp7 or the NC domain of Gag described in the text are indicated by a blue ellipse.

The HIV-1 Gag (group-specific antigen) protein coordinates all major steps in virion assembly. Gag comprises three ordered domains, namely the matrix (MA), the capsid (CA) and the nucleocapsid (NC) domains, which are connected by two linkers, p2 (also named SP1) and p1 (also named SP2) (Figure 1A). p6 was shown to be structured under membranous solution conditions [1]; however, p1–p6 was shown to be mostly disordered in the context of the C-terminal (Cter) part of Gag and to present regions that transiently adopt α-helical structures [2]. HIV-1 Gag molecules exist as monomers or low-order multimers in the cell cytosol and only form higher-order multimers after binding to the plasma membrane [3], nucleating the assembly of thousands of Gag molecules into an immature virion [4]. Small numbers of Gag molecules selectively recruit a dimeric, unspliced viral RNA genome (gRNA) selected from a pool of excess cellular RNAs and spliced viral mRNAs [4,5,6,7,8,9] through the binding of the NC domain to the 5′-leader of gRNA (Figure 1B) [10,11,12]. The formation of infectious HIV-1 particles is triggered by the sequential proteolysis of Gag polyprotein by the viral protease [13,14]. During or shortly after the budding of the particle, the HIV-1 protease cleaves Gag to trigger HIV-1 maturation [15]. Three different forms of NC appear subsequently (Figure 1A). The first cleavage by the HIV-1 protease occurs between MA-CA-p2 and NC-p1–p6, thereby liberating NCp15. The second cleavage frees NCp9 (NC-p1), whereas the last one releases the NC domain itself, which constitutes the final maturation form (NCp7). The optimal RNA chaperone activity of the NC domain, essential notably during reverse transcription (RT) [16,17,18,19,20,21,22,23,24], is observed for the mature form of NC. NCp15-containing virions are noninfectious [25,26,27,28], and the cleavage between p1 and p6 is therefore critical. The ability of the protease to achieve ordered Gag processing at specific cleavage sites, which display little to no sequence identity, primarily originates from the conformational dynamics of the protease flaps that cover its active site and that are involved in substrate recognition [29]. The modulation of the flap opening through protease/Gag interactions finely tunes the lifetime of the productive complex and hence the likelihood of Gag proteolysis.

Both MA and NC domains within Gag can bind to RNA in the cytoplasm [9,30]. However, the NC domain within Gag drives gRNA packaging by binding to the packaging signal Ψ. In contrast, MA binds a subset of tRNAs in the cytosol, which regulates MA and Gag-membrane binding [31,32]. Global changes in the RNA-binding specificity of Gag regulate virion synthesis. Before and after virion assembly and maturation, the NC within Gag preferentially binds to the Ψ region [6,33,34,35] and Rev Response Elements (RRE) of the gRNA [31]. The RNA-binding properties of NC in its mature forms hence resemble that of unassembled Gag in the cytosol. During assembly and budding, the NC within Gag nonspecifically binds to many sites on the gRNA [31], facilitating genome packaging. After assembly, the release step is mediated by the cellular ESCRT machinery, notably TSG101 and ALIX proteins [36,37,38,39,40,41]. For both TSG101 and ALIX, p6 bears the strongest interaction sites within Gag, but the NC cooperates with p6 for their binding and for promoting virus budding [42,43,44]. The coordination between protease activation, virion assembly and budding is critical and ensures the infectivity of progeny virions [15,42].

NC is a nucleic acid (NA)-binding protein that plays a role in almost all stages of the HIV-1 replication cycle: dimerization of gRNA, gRNA packaging [9], assembly of virions, hybridization of the primer tRNA^Lys^_3_ to the PBS [18,45,46,47], strand-transfer steps during RT [48,49,50] and integration of proviral DNA (for a review, see [51]). Many of these functions involve a structural reorganization of the NA structure. NC chaperones these rearrangements, leading to more thermodynamically stable structures [23]. The mature form of NC, NCp7, is a small protein of 55 residues, very basic (pI = 9.9), which contains two domains, each structured around a Zn^2+^ ion (Figure 1A), named zinc-knuckle (ZK) domains, with the consensus sequence: CX_2_CX_4_HX_4_C. The electrostatic interactions represent an essential component of NC interactions with NAs, and NC binding is indeed dramatically dependent on the ionic strength [52,53,54,55,56]. Nevertheless, some NC interactions with NAs are more specific, with G-rich single-stranded sequences flanked by stable helices being the preferred sequence binding sites for NCp7 [20,31,57,58,59,60,61,62,63].

During the viral cycle, the nucleocapsid domain exists first within the Gag protein, then within NCp15 and NCp9 during the maturation of Gag by the viral protease, and as NCp7 in the mature form. This review focuses on the NA-binding properties of NC in its different forms of maturation.

## 2. NC Domain in Its Immature States (Gag, NCp15, NCp9)

### 2.1. Structure of the Packaging Signal and Binding of NC

During the HIV-1 viral cycle, viral RNA transcripts function as mRNAs: spliced transcripts encode the viral envelope and accessory proteins, whereas unspliced transcripts produce the Gag and Gag-Pol polyproteins by regulated frameshifting during translation. Some unspliced transcripts do not function as mRNAs but are instead selected for packaging into assembling virions as dimers of gRNA [5,6,64,65], a dimer being necessary for strand transfer-mediated recombination during RT [66]. Since the discovery of HIV-1, numerous studies have focused on the selection of a dimeric gRNA among cellular and viral RNAs, which still generates multiple debates in the literature. The two main questions regarding this process are: (1) the formation/structure of the gRNA dimer through the DIS/DIS interaction in the 5′-leader of gRNA, and (2) the sequence and structure determinants of the packaging signal in the 5′-leader. Both questions involve NC-mediated binding of Gag to the 5′-leader of gRNA. The binding of Gag at the level of the DIS sequence, notably its internal loop, is responsible for its slightly higher capacity, when compared to NCp7, to recruit gRNA versus spliced HIV-1 RNAs (Figure 1B, site 1) [35,67,68]. In cellulo, Gag recognizes particular motifs such as Rev Response Element (RRE) and the packaging signal (including SL1 and SL3) [31,69]. Recent studies involving a series of impressive NMR studies [6,7,62,64,70,71,72] have shown outstanding results using smart labeling strategies in order to answer these questions [73,74,75]. In these studies, the binding of NCp7, used as a substitute for Gag in the context of gRNA dimerization and its selective packaging, was extensively studied. A detailed description of these studies is beyond the scope of this review, but we chose to present the major results related to the binding of NC.

For gRNA dimerization, the dimer formation is promoted by the base-pairing of the AUG region (gag start codon, previously known as SL4 hairpin loop) with the U5 sequence (Figure 1B) [7,71]. The dimerization process appears to expose numerous NC binding sites in the 5′-leader region. The gRNA and mRNA diversification of functions was initially thought to be modulated by a riboswitch-like mechanism, in which the role of a single RNA transcript was modulated by changes in its structure [7,76,77]. It now seems that transcript dimerization and fate are controlled at the level of transcription by heterogeneous start site usage. Indeed, transcripts that begin with two or three sequential 5′-guanosines form monomers in vitro and are retained in cells for splicing and/or translation. In contrast, those starting with a single 5′-guanosine form dimers and are selectively packaged into virions [64,78,79].

For gRNA packaging, the HIV-1 packaging signal is located within the 5′-leader of the gRNA [6,11,65], which is among the most conserved regions of the viral RNA [80,81] (Figure 1B). Early efforts to identify HIV-1 RNA packaging determinants within the 5′-leader identified stretches of nucleotides surrounding and including the Ψ hairpin (Figure 1B, site 2) that, if deleted, resulted in severe HIV-1 packaging defects [10,82,83]. The Ψ hairpin was thus proposed to be the major RNA packaging determinant. However, mutations in the GGAG loop of Ψ abolished NC binding but did not significantly affect RNA packaging [6,84], and it soon became clear that regions other than the apical loop of the Ψ hairpin were important for RNA packaging [65,70,84,85,86,87]. A region of the leader sufficient for packaging heterologous vectors into assembling virions was recently identified [70], and was shown to adopt a tandem three-way junction structure [72]. Besides, the conserved [UUUU]:[GGAG] element, located in the stem of the Ψ hairpin (Figure 1B) and adjacent to this tandem three-way junction structure, was shown to be critical both for high-affinity NC binding and competitive RNA packaging [62]. The HIV-1 leader binds approximately two dozen copies of the NC protein with affinities ranging from ∼40 nM to 1.4 μM. Each of the conserved [UUUU]:[GGAG] elements in the dimeric gRNA binds two NC molecules with affinities (∼40 nM) approximately one order of magnitude greater than those of other NC binding sites within the encapsidation-promoting region of the viral leader, and more than 30 times tighter than NC binding sites in other regions of the leader [62]. Binding to the four highest-affinity sites occurs with endothermic energetics attributed to NC-induced localized RNA melting, possibly helix melting at the level of the [UUUU]:[GGAG] element that presents a labile helical structure in the absence of NC (Figure 1B, site 3). Mutations that stabilize these sites inhibit NC binding in vitro and RNA packaging in transfected cells [62]. The absence of this [UUUU]:[GGAG] element in spliced viral transcripts is proposed to contribute to the selective packaging of the full-length gRNA. These findings appear consistent with chemical probing studies showing that residues of [UUUU]:[GGAG] were resistant to RNase V1 cleavage in the absence of NC but exhibited an enhanced chemical accessibility after Gag/NC binding [35,88].

Altogether, these studies underline the crucial role of unpaired guanines in the dimerization and packaging processes involved in the high-affinity binding to gRNA of NC within Gag.

### 2.2. Investigations of Gag, NCp15, NCp9 Structures and Dynamics, and NA-Binding Properties

The structural investigation of the full-length HIV-1 Gag protein has so far been prevented by its low solubility and its extreme sensitivity to proteolysis [89]. To date, only a Gag construct lacking p1 and p6 was amenable to NMR, and the structured domains within Gag (MA, CA and NC) were shown to retain the same fold as the isolated domains [90]. The structured domains of Gag reorient semi-independently from each other through p2 and p1 that are unstructured and that connect the structural domains. In this study, Gag exists as a dynamic equilibrium between monomers and dimers and shows a strong interaction with DNA through the NC domain and a weak secondary interaction through MA. They also confirmed that the proteolytic cleavage at site 1 (Figure 1A) was accelerated in the presence of single-stranded DNA, with no evidence of interaction between p2 and DNA, suggesting that substrate accessibility rather than substrate conformation constituted the rate limiting step for Gag proteolysis.

The affinities for NAs of NCp7 (NC), NCp9 (NC-p1) and NCp15 (NC-p1-p6), the Cter part of HIV-1 Gag, were shown to be similar [2,91]. However, distinct NA-binding properties were described concerning the kinetics of association/dissociation with NAs and the thermodynamics of interaction with NA. Slower NCp15 dissociation kinetics were observed when compared to NCp9 and NCp7, in line with the poorer NA-chaperone activity and NA-aggregation capacity of NCp15 [91]. More recently, the structure and dynamics of the Cter part of HIV-1 Gag were investigated by exploring the conformational landscape of the different stages of maturation of NC (NCp7, NCp9 and NCp15) using ^15^N relaxation measurements [2]. For the three proteins, the two ZKs define two well-structured regions separated by a more flexible linker, showing that the global folding and dynamics of the NC domain is conserved during maturation. However, relative to NCp7 and NCp9, the N-terminal (Nter) part of NCp15 is more rigid, while residues of the ZKs and the linker are globally more flexible [2]. p1–p6 within NCp15 is disordered, but short-lived α-helices are present in p1-p6 and are promoted by long-range and transient contacts with the NC domain. Notably, p1 transiently interacts at one site comprising the ZKs through hydrophobic contacts, whereas p6 has multiple sites of interaction in the Nter part of NC, which is unfolded and positively charged, through electrostatic interactions [2]. The three proteins can bind the SL3/Ψ hairpin using the same specific interactions, and p1-p6 do not participate directly in SL3 binding. This explains why the affinities for NAs are the same for NCp7, NCp9 and NCp15. However, the precursors NCp9 and NCp15 exhibit a higher entropic penalty for the binding of SL3 than NCp7. These changes in the entropic penalty for SL3 binding are correlated with changes in the fast dynamics observed for the different states of maturation of the NC domain. Indeed, the increase in global flexibility of the ZKs and of the linker does not change the global affinity of the NC domain for SL3, but instead results in a conformational entropic penalty to SL3 binding by the precursors. This study thus provides a new picture of the structural and dynamic properties of the NC domain during the assembly, budding and maturation of virions [2]. During the assembly and budding of virions, concomitant with Gag oligomerization, the transient interactions between NC and p1–p6 become salient and responsible for (i) a higher level of p6 secondary structure favoring the recruitment of its budding partners and (ii) a higher entropic penalty to NC RNA-binding at specific sites, leading to the nonspecific RNA-binding of NC at a multitude of sites on gRNA that are necessary during assembly. The maturation by the protease cleaving off p6 and p1 from NC reverts the RNA-binding specificity of the mature NC. NCp7 no longer binds to multiple sites but binds to discrete specific sites on gRNA containing unpaired guanine. This model explains the apparent changes of the RNA-binding specificity of the NC domain during assembly and agrees with an ITC study showing that oligomerization-competent forms of Gag displayed a ∼3-times stronger binding affinity for nonspecific RNA motifs over the cytosolic specific target of NC [92]. This was not observed for oligomerization-impaired forms of Gag, and for Gag lacking p6 and capable of high-order Gag oligomerization [92]. In conclusion, p1–p6 has a role in facilitating the binding of nonspecific RNA motifs to oligomeric forms of Gag present at the moment of the assembly of the virions.

## 3. NCp7 and NA Interactions

### 3.1. NMR Structures of NCp7-NA Complexes

**Figure 2:**
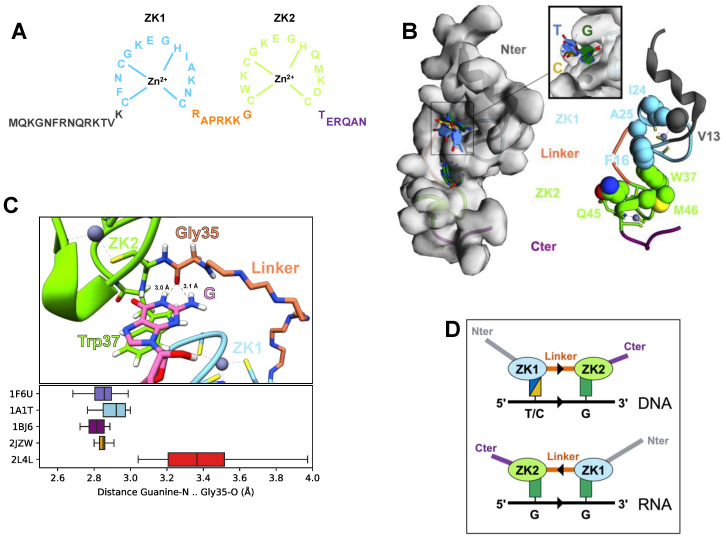
Structural determinants of NCp7-NA complexes: (**A**) Sequence of the HIV-1 NCp7 for the NL4.3 strain; (**B**) 3D structure representations of the interaction interface of NCp7 with NA. (**Left**) Surface of NCp7 in complex with the Ψ hairpin, also named SL3 (PDB: 1A1T), showing the location of the two NA-binding pockets in each ZK. The backbone trace is colored to highlight the different regions of NC. The bases bound in the pockets (shown as filled rings and colored according to the base type, see inset) are taken from all available structures of NC-NA complexes (each pocket is superposed using the C⍺ atoms of the corresponding ZK). (**Right**) The side chains lining the two NA-binding pockets are shown as Van der Waals spheres; Zn^2+^ ions are shown as gray spheres, while coordinating residues are shown as sticks. (**C**) Hydrogen bonds between the guanine bound in the ZK2 pocket and the backbone of Gly35 in NC-NA complexes. (**Top**) Interaction between the G base (pink) and the backbone of Gly35 (orange) in the NC-SL3 complex. G is stacked with the side chain of Trp37 (green). The linker is shown as the minimal backbone trace. (**Bottom**) Box plot of the distance between the backbone oxygen of Gly35 and the closest nitrogen of the Guanine bound in the ZK2 pocket, showing that the distances are compatible with a hydrogen bond in all conformations of all available NC-NA complexes. (**D**) Schematic representation of the respective polarity of NC and NA in NC-NA complexes. In NC-DNA complexes (**top**), the Nter→Cter direction of NC is parallel to the 5′→3′ direction of the DNA, whereas in NC-RNA complexes (**bottom**), the two directions are antiparallel.

To date, five structures of NCp7-NA complexes are available in the PDB and have been determined using NMR spectroscopy [58,59,60,61,93,94]. All NC-RNA complexes were obtained using the NC sequence from the NL4-3 strain (Figure 2A) and with RNA fragments that were either hairpins or a single-stranded RNA. The structures of NC-DNA complexes were solved with a truncated form of NC, lacking the first ten Nter residues, with the NC sequence from the MAL strain and with DNA hairpins (TAR, PBS) or a single-stranded DNA. Overall, the folding of the individual ZKs was the same in all complexes. A recent study has extensively analyzed these complexes, and using bioinformatic methods has quantified the interface between NC and NA in all the available 84 conformers [95]. The ZK2-NA interface was the same in all structures, while a broad diversity of ZK1-NA interfaces was observed. The reason for this diversity is not apparent and may originate from the differences in NA and/or NC sequences. The quantification of the contact areas shows that Phe16 and Arg/Lys26 in ZK1 and Trp37 in ZK2 are the most contacted residues in NC-NA complexes. Each ZK has a hydrophobic pocket for recognizing a base in a single-stranded part of the NA. The hydrophobic pockets are defined by Val13, Phe16, Ile24, Ala25 in ZK1 and Trp37, Gln45 and Met46 in ZK2 (Figure 2B). In all NCp7-NA complexes, a guanine is deeply inserted inside the hydrophobic pocket of ZK2 and interacts through π-π stacking with Trp37. ZK1 binding is more versatile, and ZK1 can make contacts with either a guanine, a thymine or a cytosine (Figure 2B). However, for the complexes with a thymine or a cytosine bound in ZK1, the base is located at the periphery of the hydrophobic pocket. In contrast, a guanine is deeply inserted into the ZK1 hydrophobic pocket (Figure 2B, inset) through π-π stacking with Phe16. In addition to π-π stacking with aromatic residues, the binding of guanines in each ZK is reinforced by hydrogen bonds between the guanine base and the backbone of several residues, including residues of the linker. For instance, in all NCp7-NA complexes, Gly35 is involved in a network of hydrogen bonds with the guanine in ZK2 (Figure 2C). These structural studies agree with fluorescence spectroscopic studies [19,52,56,96,97,98], insofar as they show that ZKs contain hydrophobic residues at adequate positions to form an ideal surface for the recognition of guanine residues located in a single-stranded section of the NA.

In all complexes, arginine and lysine residues, fifteen of which are present in full-length NCp7, are observed to participate in numerous nonspecific electrostatic interactions with the phosphodiester backbone of NA. Notably, the Nter part of NCp7, present in all NCp7-RNA complexes, is very basic and folds as a 3_10_ helical structure upon binding to the RNA. Three salt bridges, not observed in free NCp7 structures [25,99], are observed within NCp7 in several complexes: Lys33–Glu42 (between linker and ZF2), Lys38–Glu51 (within ZF2) and Lys14–Glu21 (within ZF1). These structural data are in agreement with a large number of physico-chemical studies pointing out the significant role of ionic forces in stabilizing the NC and Gag-NA complexes [33,55,96,100,101,102].

Last, the analysis of NCp7-NA structures shows that NCp7 can interact differently with the backbone riboses according to the nature of the NA [61]. Indeed, in NC-DNA complexes, ZK1 interacts with a base upstream (5′-side) of the G bound by ZK2, whereas in NC-RNA complexes, ZK1 interacts with a residue located downstream (3′-side) of this G (Figure 2D). Thus, NC can adapt to different sugar conformations to trap the G in the hydrophobic pocket of ZK2, suggesting that the binding of NCp7 to NA is driven by this interaction. This ability to recognize NA polarity is in agreement with the large differences in binding affinities measured according to the NA sequence polarity [56], as described for other single-stranded DNA-binding proteins [103].

Altogether, these structures explain the preference of NC for single-stranded NAs and for unpaired guanine and how NC can recognize either RNA or DNA.

### 3.2. Dynamics of NCp7 and NCp7-NA Complexes

Early study of the dynamics of NCp7 using ^15^N NMR relaxation demonstrated that NCp7 is in a rapid equilibrium between several conformations from weakly interacting to noninteracting ZKs [99]. The linker, the Nter and Cter parts of NCp7 are disordered and particularly flexible. In contrast, both ZKs adopt a folded and more rigid conformation and reorient independently of each other due to the linker’s flexibility. This suggests that the two ZKs could function independently from each other in the different steps of the NA recognition process.

NCp7 dynamics were further analyzed using ^15^N relaxation measurements performed at several magnetic fields [2,104], combining residual dipolar coupling (RDC), SAXS and ensemble-simulated annealing [105], using relaxation dispersion and chemical exchange saturation transfer measurements [106], as well as molecular simulations [107]. Globally, these data demonstrated that the two ZKs were not equivalent. While the overall conformational space sampled by NCp7 was large, the conformational distribution of ZK1 was distinct from that of ZK2 as a result of differential flexibility along the linker region. The largest conformational freedom was observed for Lys34 and Gly35, comparable to a hinge, located at Gly35 just before the beginning of ZK2. This feature was also observed in NCp9 and NCp15 [2]. In contrast, linker residues from Cys28 to Lys33 displayed restricted motions and numerous contacts with residues of ZK1. This differential flexibility within the linker resulted in an asymmetric motion of the ZKs, which may explain their distinct role in NA-binding despite their high sequence identity (see the following paragraphs).

**Figure 3:**
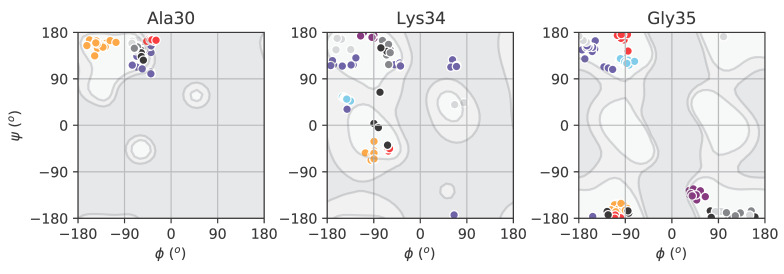
Ramachandran plot for selected linker residues. The φ and ψ values for Ala30 (**left**), Lys34 (**middle**) and Gly35 (**right**) are superposed to the probability distribution of the same residue in globular proteins taken from high-resolution structures in the PDB (https://www.rcsb.org/), where lighter shades of gray indicate a higher probability of occurrence. Ala30 is shown as a reference for poor variation in free NC. Backbone dihedral angles were calculated on the following PDB structures: 5l1R [105] (NCp7, grey, 21 conformers); 1A1T [59] (NCp7-SL3, sky blue, 25 conformers); 1F6U [60] (SL2-NC, slate blue, 20 conformers); 2L4L [61] (cTAR-NC, red, 10 conformers); 2JZW [58] (NCp7-PBS, orange, 19 conformers); 1BJ6 [93] (NCp7-d(ACGCC), purple, 10 conformers). The 21 conformers of 5I1R are further subdivided according to the definitions given in the original paper: cluster 1 (grey, ~40%), cluster 2 (dark grey, ~50%), cluster 3 (light grey, ~10%).

NCp7-NA complexes globally exhibit a loss of flexibility upon binding for both the NA and NCp7 [2,97,98,108,109]. To compare the flexibility within the linker in NCp7 and NCp7-NA complexes, we analyzed the backbone dihedral angles (φ and ψ) of linker residues (Ala30 to Gly35) in all the available structures (Figure 3). In the unbound NCp7 [105], Lys34 and Gly35 exhibited by far the largest backbone dihedral fluctuations among the residues in the linker (Figure 3, grey points), confirming the role of these two residues as a hinge for ZK motions. In particular, this backbone flexibility seems to involve the ψ angle of Lys34 and the φ angle of Gly35. Binding to NAs significantly reduces the conformational freedom of Lys34 and Gly35, narrowing the range of accessible φ and ψ values, particularly for Gly35, which is involved in the guanine binding (Figure 2C). It is tempting to speculate that directing the backbone flexibility at the level of a few degrees of freedom involving the backbone of Gly35 could create a molecular switch that would entail a more rigid structure of NC upon NA binding.

### 3.3. Conclusions

Altogether, the structural and dynamic data discussed here highlight several aspects of NC-NAs interactions: 1) ZK2 interacts with unpaired guanines, the preferred substrate of NC, and is more prone to do so than ZK1; 2) NC orients on NA chains with a definite polarity, depending on the nature of the NA (DNA or RNA); 3) the dynamics of the two ZKs are remarkably different, due to their different localizations in the sequence rather than to their slightly different compositions in amino acids. These biophysical properties explain a number of observations reported in the literature, and the next paragraph is devoted to this point.

## 4. The Two ZKs Are Not Equivalent

### 4.1. Biochemical and Biological Evidence

Numerous studies focused on the distinct roles of ZKs in the various steps of the replication cycle. In Table 1, we have summarized the modifications or mutations incorporated in NC and the phenotype observed for in vivo studies, or the defects observed in one step of the viral cycle for in vitro studies. 

Initial studies on NC modifications covered: (i) alterations in the coordination array of each Zn^2+^ ion (mutation of C to S or H and H to C or A) [10,12,111,112,113,114,115,126]; (ii) swapped mutants that exchanged the position between ZK1 and ZK2 (ZK2-ZK1) or duplication of one ZK (ZK1-ZK1 or ZK2-ZK2) [115]; (iii) modifications of the basic residues located inside and/or outside of the two ZKs [110,122,123,127]. In most of these studies, plasmids containing the NC-mutated HIV genome were transfected in cells, and viral particles were then extracted and analyzed after several days of post-transfection cultures [10,12,110,115]. The major result from these studies was that both ZKs were required for an optimal RNA packaging and efficient viral infectivity. However, alterations in ZK1 provoked more deleterious defects than in ZK2 [12]. Interestingly, these studies were crucial to identifying that NC was involved in an early step of the replication cycle occurring before the RNA packaging: subsequently, it was found that this step was the reverse transcription (RT) and that an NC mutant able to partially perform RNA packaging could be totally unable to perform RT, leading to noninfectious viruses [12,112].

The discovery of the involvement of NCp7 or NCp9 in gRNA dimerization and in different steps of RT, such as the initiation of RT through the hybridization of tRNA^Lys^_3_ to the PBS in the 5′-leader of gRNA and the stimulation of the two strand-transfers during RT [16,23,24,98,128,129], fueled further investigations of the NA chaperone capability of NC, as well as of its NA-binding properties. Indeed, effective binding is a necessary, but not sufficient, condition for NA-chaperone activity. Modifications of the two ZKs were found to affect the RNA-packaging and NA-chaperone activities of NC (strand-transfer, self-priming, NA annealing and NA-helix destabilization) differently. Pioneering studies have shown that ZK1 is absolutely required for RNA annealing, such as HIV-1 gRNA dimerization or annealing of tRNA^Lys^_3_ to the PBS [45,130]. ZK2-ZK2 and ZK2-ZK1 constructs of NCp7 did not exhibit any chaperone activity, whereas ZK1-ZK1 showed a satisfying (but nonoptimal) chaperone activity [100]. ZK1 is thus more critical for chaperone activity than ZK2, and must be located in the Nter part of NC for a correct chaperone activity [100,116,117]. ZK2 is, however, necessary to increase the NC annealing rate [116]. In addition, the ZK1-ZK1 construct is a weaker binder than NCp7 but performs better than the ZK2-ZK2 and ZK2-ZK1 mutants [131,132,133]. More recently, a peptide comprising the first 35 Nter residues of NC, NC(1–35), was shown to be necessary and sufficient for NA-chaperone activity in vitro [118], highlighting the critical role of ZK1 in this context. NC(1–35) displays a binding affinity for NAs similar to that of NC, whereas NC(1–28) (further lacking the linker) and NC(29–55) (containing just the linker and ZK2) have decreased affinities [118]. NC(1–35) can also recognize the packaging signal Ψ, while NC(29–55) cannot [119]. Similar ZK constructs produced in the context of the GagΔp6 precursor gave totally different results in assays of Ψ binding in a 1M concentration of NaCl, thus favoring the nonelectrostatic component of the interaction [102]. While the construct with ZK2 has almost the same affinity for Ψ as NC, those with ZK1 (i.e., ΔZK2) exhibit a dramatic decrease of three orders of magnitude [102], in agreement with previous data [55]. The contradictory results of these studies underline the importance of the conditions used to analyze the contribution of the two ZKs to NA binding affinities. A critical point could also lie in the ability of the linker residues to interact with ZK1 residues. Indeed, such interactions have been proposed as exerting a significant influence on NA binding [104,105,134]. A comprehensive study of NC using polyetheno-adenylic acid showed that NA binding stems more from ZK1 than from ZK2, notably with two lysines from ZK1 involved in electrostatic contacts with NA instead of only one for ZK2 [55]. These results are rather well corroborated by the structure of the NC-SL3 Ψ complex [59]. Additionally, when working at a high salt concentration (1M NaCl) to focus on the nonelectrostatic contribution of NA-binding, results are similar to those obtained in the context of Gag [102].

The molecular basis of the ability of ZK1 to better destabilize the NA secondary structure, i.e., helix unwinding ability, was investigated in several studies [55,97,116,131,135]. Point mutations in ZK1 to reproduce the sequence of ZK2 show that the I24Q mutation impairs helix unwinding, while N27D alters both RNA binding and helix unwinding [135]. The more hydrophobic nature of ZK1, relative to ZK2, was generally proposed to explain the higher helix-unwinding ability of ZK1 [55,97,116,131,135]. In the case of the PBS (-), whose stem’s destabilization by NC has been established [58], the complex structure shows that the stem is in contact with hydrophobic residues of ZK1, in particular Val13, Phe16, Thr24 and Ala25 [58,104]. In all structures of NC in a complex with hairpins, whether RNA or DNA [58,59,60], the stem of the hairpin interacts with the hydrophobic residues of ZK1, but not with those of ZK2 (Figure 2B), corroborating the implication of ZK1 in the helix-unwinding activity.

Biophysical methods, like optical tweezers and FRET, were also extensively used to probe the NA chaperone activity in response to different alterations of the ZKs [117,120,131,136]. These studies confirmed the major role of ZK1 in the NA-destabilization activity of NC, its necessity to be located at the Nter part of NC and the critical role of Trp37 in ZK2 [131,135,137]. This role of Trp37 was further described in several publications [97,109,120,121,131], showing the interdependence of the two ZKs in this activity. The mutation of Phe16, the aromatic residue in ZK1 that occupies the same position as Trp37 in ZK2, to a nonaromatic residue (Ala or Leu) also appears to reduce the chaperone activity of NC. Still, these effects are less severe than for Trp37 [120,131]. These aromatic residues, Phe16 and Trp37, also play a crucial role in NA binding: mutating either of these to a nonaromatic residue strongly affects the NA-binding affinities of NC [56,97,120,131,132,138], in agreement with NA-NC structures; again, mutating Trp37 was more deleterious than mutating Phe16 [97,120].

Altogether, this large corpus of data suggests that ZK1 plus the linker, NC(1-35), possess all the needed elements to achieve chaperone activity and NA binding, including the selective binding of Ψ [118,119]. However, the mutation of Trp37 in ZK2 to a nonaromatic residue dramatically alters both the NA-binding and chaperone activities [55,120,131]. This apparent contradiction may be reconciled in light of the recent analysis of NC-RNA complexes [95], where both ZKs interact with a guanine. The largest contact area with RNA is observed for ZK1, whereas Trp37 in ZK2 presents the highest individual contribution. The following picture thus emerges: ZK1, due to its larger contact area both in terms of electrostatic and hydrophobic interactions with NA, is necessary in order to provide NA-NC complexes with a sufficient affinity to interact, and the proper interaction of Trp37 with a guanine is required to obtain a complex with an optimal affinity and specificity. Additionally, our analysis of the dynamic properties of NC-NA complexes (see Section 3.2) suggests that guanine binding in ZK2 leads to a conformational switch in the linker, reducing its flexibility at the level of the Gly35 hinge and thus potentially modifying the ZK1 interaction properties.

A last example of the difference between the two ZKs can be found in the studies that uncovered, upon deletion of one or two ZKs, an unexpected premature viral DNA synthesis starting in virus-producing cells and leading to the production of noninfectious particles containing a high level of viral DNA [124,139]. A substantial difference is observed in the quantity of viral DNA measured in virions depending on which ZK was deleted, with the most considerable effect marked for the deletion of ZK2 [124]. The fine analysis of these mechanisms points to the importance of NC and of its adjacent domains p1 and p6 in the precise timing of the budding events; the perturbation of this schedule leads to premature RT in infected cells [42,124,140]. These latter events involve interactions of NC and p6 with cellular factors (TSG101, ALIX) [14,15,42,43,44]. Interestingly, the deletion of essential parts of the packaging signal Ψ (SL1, SL3) also results in premature RT, like the deletion of ZK2, suggesting that direct contact between SL1-SL3 and ZK2 could be needed to restrict the premature RT process and trigger the correct succession of events leading to the budding of virions [15]. These data thus suggest a specific recognition between NC and gRNA in the control of the budding process, complementing the role of cellular proteins of the ESCRT family, with the optimal recognition requiring the presence of ZK2 [14,42].

Interestingly, a recent study examining the contribution of each ZK to the accumulation of Gag-gRNA complexes at the plasma membrane identified ZK2 as the major contributor in this task, underlining one more time the importance of ZK2 in the recruitment of the ribonucleoprotein complexes involved in the assembly process [125].

### 4.2. Conclusions about the Functions of the Two ZKs

**Figure 4:**
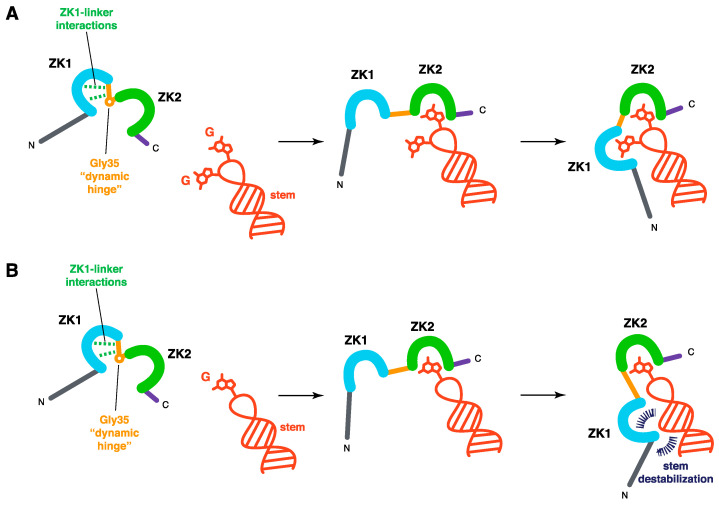
Schematic representation of the task specialization of each ZK upon binding to NA. The NA fragment is in red, NC ZKs are in cyan (ZK1) and green (ZK2), NC Nter and Cter tails are in grey and purple, respectively, and the inter-ZK linker is in orange. In the NA-free form of NC, interactions between the more rigid part of the linker and ZK1 are represented with dashed lines in green. In addition, within the linker, the Gly35 dynamic hinge is shown (see paragraph 3.2 and Figure 3). This dynamic hinge is not retained in NA-bound forms of NC, Gly35 being directly involved in the guanine recognition within ZK2 (see Figure 2C). (**A**) Situation 1, with two accessible guanines. The ZK1-linker interactions render ZK1 less accessible than ZK2. Thus, ZK2 binds first to an accessible guanine, and ZK1 binds the remaining guanine. The stem is not destabilized. (**B**) Situation 2, with only one accessible guanine. Similarly, ZK2 binds first to the unique accessible guanine, and ZK1 remains free to contact the stem via its large hydrophobic platform. The stem is destabilized (in dark blue).

In conclusion, the two ZKs have different functions in the processes involving NC-NA interactions, namely the selective packaging of the gRNA, the NC chaperone activity and the control of the late RT process in the final steps of the cell infection. ZK1 appears necessary for high-affinity binding and is more critical in the chaperone activity than ZK2. Trp37 in ZK2 is crucial for the specific recognition of unpaired guanine. Notably, NC (1–35) appears necessary and sufficient to achieve most of the essential functions of NC; however, in NCp7, the mutation of Trp37 leads to a loss of these functions. The dynamics of the two ZKs are also different, since the interactions between the linker and ZK1 and the variations in flexibility along the linker render the motions of the two ZKs asymmetric. The literature is thus entirely consistent with a two-step model, in which Trp37 in ZK2 is first required to bind one unpaired guanine, followed by the binding of ZK1 to NA to produce a more stable complex and to allow NA stem destabilization if needed (Figure 4).

This model assumes that, in NCp7, ZK1 is not as accessible as ZK2 for RNA binding. The ZK1-linker contacts would prevent the ZK1 domain from interacting with unpaired guanines, whereas the ZK2 domain would be more accessible and competent to interact with unpaired guanines. In contrast, ZK1, with its sizeable hydrophobic plateau, would be able to destabilize the double-stranded regions adjacent to the guanines bound by ZK2. NMR data support the notion that the two ZKs are not equivalent and present different hydrodynamic properties. Moreover, small molecules able to eject zinc ions preferentially target ZK2 rather than ZK1 [106,141,142,143,144]. Similarly, alkylating agents, such as N-ethylmaleimide, react preferentially with the thiolates of the cysteines in ZK2. Interestingly, for N-ethylmaleimide, it was shown that the reactivity is different for the isolated ZKs, with a higher reactivity for isolated ZK1 compared to ZK2, in contrast to the results observed within the native NCp7 [145]. The linker residues and the internal dynamics of NC therefore regulate the different functions of the two ZKs that are required for an optimal chaperone activity.

## 5. Conclusions

NCp7, the final maturation state of the NC domain, is the most studied form of this domain since the discovery of HIV-1. It is now clear that the two ZKs are not equivalent, due to their position in the NC sequence, the interactions between the linker and ZK1, and the variations in flexibility along the linker. Recent studies demonstrate that the NC domain of Gag, NCp15 and NCp9 behaves exactly the same as in the mature form. However, the interactions between p1–p6 and the ZKs modulate the dynamics of the ZKs and finely tune the interaction of the NC domain with NAs. These interactions can explain how the NC domain can change its NA-binding preferences during the assembly and budding of virions.

## Figures and Tables

**Table 1:** Implication of NC protein modifications or mutations on the phenotype and/or defects of the viral cycle.

Modifications/Mutations	Phenotype/Defects	Conclusions	References
Cx->Sx or Hy->Ay	Defect in RNA packaging, assembly not correct, defects in NA annealing	More deleterious effects for mutations in ZK1	[12,110,111]
Cx->Hx and Hy->Cy	Normal RNA packaging but virus noninfectious	More deleterious effects for mutations in ZK1	[112,113,114]
ZK2-ZK1 ZK1-ZK1 ZK2-ZK2	Defect in strand-transfer and self-priming reaction during RT	ZK1 must be in the first position, a major role of ZK1 for destabilization activity of NC	[100,115]
ZK2-ZK1 ZK1-ZK1 ZK2-ZK2	Defect in NA annealing	ZK1 must be in the first position, a major role of ZK1 for destabilization activity of NC	[116]
ZK2-ZK1 ZK1-ZK1 ZK2-ZK2	Defects in DNA stretching ability as a test of chaperone activity	ZK1 must be in the first position, a major role of ZK1 for destabilization activity of NC	[117]
NC(1–35)	Sufficient for in vitro chaperone activity	Critical need of ZK1 for chaperone activity	[118]
NC(29–55)	Not able to recognize packaging signal, low affinity for NA	Critical need of ZK1 for interaction with NA	[118,119]
Mutation of Trp37 in ZK2 or Phe16 in ZK1	Defect in NC chaperone activity, NA-binding strongly affected	Critical role of Trp37 in ZK2, less critical for Phe16 in ZK1	[120]
Basic residues	Inhibition of gRNA dimerization, reduction of gRNA packaging, strong effect on virus assembly, reduction of infectivity	Critical for RNA packaging, optimal chaperone activity and infectivity	[101,121,122,123]
Deletion of the linker	Decrease of NA-binding affinity	Linker contributes to NA-binding and chaperone activity of NC	[118]
Deletion of ZK1 and/or ZK2 within Gag	Production of viral particles containing DNA	ZK2 more important than ZK1 to block premature RT	[42,124]
Deletion of ZK1 and/or ZK2 within Gag	Loss of affinity in Gag-gRNA complexes	ZK2 more important to ensure Gag-gRNA complexes of high affinities	[102]
Deletion of ZK1 and/or ZK2 within Gag	Recruitment of Gag-gRNA complexes at the plasma membrane (PM)	ZK2 more important for the accumulation of ribonucleoprotein complexes at the PM	[125]
